# Real-World Comparison of PD-1 and PD-L1 Inhibitor Monotherapy in Metastatic Non-Small Cell Lung Cancer with High PD-L1 Expression

**DOI:** 10.3390/cancers18071153

**Published:** 2026-04-03

**Authors:** Filip Marković, Zlatan Bojić, Mihailo Stjepanović, Milica Kontić

**Affiliations:** 1 Clinic for Pulmonology, University Clinical Centre of Serbia, 11000 Belgrade, Serbia; bojic.zlatan@gmail.com (Z.B.); milicakontic@yahoo.com (M.K.); 2 Faculty of Medicine, University of Belgrade, 11000 Belgrade, Serbia

**Keywords:** immune checkpoint inhibitor, non-small cell lung cancer, pembrolizumab, atezolizumab, real-world data

## Abstract

**Background:** Immune checkpoint inhibitor monotherapy targeting the PD-1/PD-L1 axis is a standard first-line treatment option for patients with metastatic non-small cell lung cancer (NSCLC) and high PD-L1 expression. However, comparative real-world data evaluating PD-1 versus PD-L1 inhibitor monotherapy in this population remain limited. **Methods:** We conducted a retrospective, single-center real-world analysis of patients with metastatic NSCLC and high PD-L1 expression who initiated first-line immune checkpoint inhibitor monotherapy between May 2024 and January 2025. Patients were treated with either pembrolizumab (PD-1 inhibitor) or atezolizumab (PD-L1 inhibitor) as monotherapy. Progression-free survival (PFS) was estimated using the Kaplan–Meier method and compared using the log-rank test. Univariable and multivariable Cox proportional hazards regression models were applied to identify clinical factors associated with PFS. **Results:** A total of 125 patients were included, of whom 73 received pembrolizumab and 52 received atezolizumab. The median PFS was numerically longer in patients treated with pembrolizumab compared with atezolizumab (9.93 vs. 5.33 months), although this difference did not reach statistical significance (HR 1.14, 95% CI 0.74–1.74; *p* = 0.559). Poor performance status (ECOG PS 2) was independently associated with inferior PFS (HR 3.22, 95% CI 2.02–5.12), whereas very high PD-L1 expression (TPS 90–100%) was independently associated with improved PFS (HR 0.57, 95% CI 0.35–0.93). **Conclusions:** In this real-world cohort, pembrolizumab was associated with numerically longer PFS than atezolizumab; however, this difference was not statistically significant. Outcomes were primarily driven by baseline clinical factors, particularly performance status and the degree of PD-L1 expression. Larger cohorts with longer follow-up are warranted to clarify potential differences and assess long-term outcomes.

## Simple Summary

Patients with metastatic non-small cell lung cancer (NSCLC) and high PD-L1 expression can be treated with an immune checkpoint inhibitor monotherapy as first-line therapy. Pembrolizumab and atezolizumab are both approved and reimbursed options in this setting, but comparative real-world data are limited. In this single-center real-world study, we compared outcomes of patients treated with pembrolizumab or atezolizumab monotherapy. Median progression-free survival was numerically longer in patients treated with pembrolizumab than in those treated with atezolizumab (9.9 vs. 5.3 months), although this difference was not statistically significant. Clinical outcomes were more strongly influenced by patient-related factors than by treatment choice. Poor performance status was associated with worse progression-free survival, while very high PD-L1 expression (90–100%) was associated with better outcomes. These findings suggest comparable effectiveness of pembrolizumab and atezolizumab in routine clinical practice; however, studies with longer follow-up and larger patient populations are needed to confirm these results.

## 1. Introduction

Lung cancer continues to represent one of the most significant oncologic burdens worldwide and remains the primary cause of cancer-related death [1]. Despite advances in screening and therapeutic strategies, prognosis remains poor, particularly for patients diagnosed at advanced stages [1]. Non-small cell lung cancer (NSCLC) accounts for approximately 80–85% of all lung cancer cases, with the majority of patients presenting with advanced or metastatic disease at the time of diagnosis [2,3].

Historically, platinum-based doublet chemotherapy represented the standard first-line approach for metastatic NSCLC, providing limited survival benefit and substantial toxicity, particularly in older and comorbid patients. The integration of immune checkpoint inhibitors has expanded treatment options and enabled chemotherapy-sparing strategies in selected populations [4]. Nevertheless, important gaps remain regarding the comparative effectiveness of different immune checkpoint inhibitor classes in routine clinical practice, where patient selection, comorbidities, and access to therapies may differ from randomized trials.

The emergence of immune checkpoint inhibition has led to a paradigm shift in the management of metastatic NSCLC. By modulating immune escape mechanisms—most notably through inhibition of the PD-1/PD-L1 signaling pathway—these agents have demonstrated meaningful and durable clinical benefit in selected patient populations. In patients with metastatic NSCLC characterized by high PD-L1 expression and the absence of actionable driver alterations, immune checkpoint inhibitor-based therapy has become a central component of first-line treatment [4]. Current ESMO Clinical Practice Guidelines endorse multiple therapeutic approaches in this setting, including immune checkpoint inhibitor monotherapy and combination regimens with chemotherapy and with anti-angiogenic agents, with treatment selection informed by clinical characteristics, disease burden, and patient performance status [4].

The PD-1/PD-L1 pathway is a central mechanism of tumor immune evasion in NSCLC. PD-1 is expressed on activated T cells, while PD-L1 may be upregulated on tumor cells and immune cells within the tumor microenvironment [5,6,7]. Binding of PD-L1 to PD-1 attenuates T-cell signaling and promotes functional exhaustion, thereby limiting antitumor immunity [5]. Therapeutic antibodies targeting PD-1 (e.g., pembrolizumab) or PD-L1 (e.g., atezolizumab) can restore T-cell activity; however, biological differences between PD-1 and PD-L1 blockade may theoretically translate into differences in effectiveness or toxicity, which remains insufficiently characterized in real-world settings [6].

However, despite broadly harmonized guideline recommendations, real-world treatment patterns vary considerably across countries and healthcare systems. Differences in reimbursement policies, drug availability, and access to molecular diagnostics may influence which treatment strategies are implemented in routine practice, particularly in resource-limited settings [8,9]. As a result, outcomes observed in randomized controlled trials may not be fully reproducible in unselected real-world populations, underscoring the importance of real-world evidence to complement clinical trial data.

In this context, immune checkpoint inhibitor monotherapy targeting the PD-1 or PD-L1 pathway has represented a widely used first-line approach for patients with high PD-L1-expressing metastatic NSCLC [10]. While both PD-1 and PD-L1 inhibitors are supported by clinical trial evidence and international guidelines, comparative data evaluating their real-world effectiveness remain limited. Moreover, it remains unclear whether outcomes differ between PD-1 and PD-L1 blockade when applied in routine clinical practice, or which clinical factors most strongly influence treatment outcomes in this population.

To address these gaps, we conducted a real-world, single-center retrospective analysis evaluating first-line immune checkpoint inhibitor monotherapy in patients with metastatic non-small cell lung cancer and high PD-L1 expression. The analysis focused on agents targeting the PD-1 and PD-L1 pathways and sought to explore treatment effectiveness in routine clinical practice, as well as to examine clinical factors associated with outcomes in the overall study population.

## 2. Materials and Methods

This study included patients with histologically confirmed metastatic non-small cell lung cancer with high PD-L1 expression who initiated first-line immunotherapy with either atezolizumab or pembrolizumab monotherapy. Pembrolizumab has been approved and reimbursed by the national health insurance system of the Republic of Serbia for the treatment of metastatic non-small cell lung cancer with PD-L1 tumor proportion score ≥ 50% since 2018. Atezolizumab was subsequently approved and included in the national reimbursement list for the same indication in May 2024. The study period covered patients who started treatment between May 2024, when atezolizumab became available for this indication at our institution, and January 2025. Treatment selection (pembrolizumab vs. atezolizumab) was not randomized. The choice was primarily driven by drug availability/reimbursement timing, treating physician preference, and individual clinical considerations.

Patients were followed up with until 31 December 2025. This was a retrospective, single-center study conducted at an academic institution in Serbia.

All patients underwent routine PD-L1 testing performed on formalin-fixed, paraffin-embedded histology or cytology samples using PD-L1 monoclonal antibodies (22C3 clone by DAKO, Glostrup, Denmark, or SP263 assay by Ventana Medical Systems, Oro Valley, AZ, USA) prior to initiating the first line of treatment. Patients were tested for EGFR mutations by Cobas^®^ EGFR Mutation Test v2 (Roche Molecular Systems, Inc., Pleasanton, CA, USA) and ALK rearrangements by immunohistochemistry prior to first-line treatment initiation.

Patients with detected driver oncogenes were excluded, so none of the included patients had a known driver oncogene. All patients were followed and assessed for best response to therapy according to RECIST v1.1 criteria according to local practice [11]. Toxicity was graded according to the Common Terminology Criteria for Adverse Events (CTCAE) v5.0 [12].

All consecutive patients with metastatic NSCLC and PD-L1 TPS ≥ 50% who initiated first-line immune checkpoint inhibitor monotherapy (pembrolizumab or atezolizumab) during the study period were retrospectively identified from institutional electronic health records and included if they met the predefined eligibility criteria.

Concomitant medications were not systematically captured beyond routine clinical documentation; therefore, residual confounding related to concomitant treatments cannot be excluded.

### 2.1. Ethics Approval

Data on patients with lung cancer were retrospectively extracted from institutional electronic health records, including demographic, pathological, molecular, treatment, and survival data of patients diagnosed and treated at the University Clinical Center of Serbia. All data were collected and analyzed in an anonymized manner. The study was conducted in accordance with the Declaration of Helsinki and approved by the Ethics Committee of the University Clinical Center of Serbia (approval number: 1880/39: 25 December 2025).

### 2.2. Statistical Analysis

Descriptive statistics were used to summarize patient demographic and clinical characteristics. Progression-free survival (PFS) was defined as the time from initiation of immunotherapy to disease progression. Patients without a PFS event at the last follow-up were censored at that date. Median PFS was estimated using the Kaplan–Meier method and compared between groups using the log-rank test.

Univariable and multivariable Cox proportional hazards regression models were applied to estimate hazard ratios (HRs) with corresponding 95% confidence intervals (CIs). Covariates included in the univariable analysis were age, sex, smoking status (ever smoker vs. never smoker), ECOG performance status (0–1 vs. 2), presence of baseline brain metastases (yes vs. no), radiotherapy during treatment (yes vs. no), treatment group (atezolizumab vs. pembrolizumab), and the occurrence of immune-related adverse events (yes vs. no). Variables with a significance level of *p* < 0.10 in univariable analysis were entered into the multivariable model.

The association between treatment response and the treatment group (atezolizumab vs. pembrolizumab) was evaluated using the chi-square test. All statistical tests were two-sided, and a *p* value < 0.05 was considered statistically significant. Statistical analyses were performed using SPSS software, version 26 (IBM Corp., Armonk, NY, USA).

## 3. Results

A total of 125 patients with metastatic non-small cell lung cancer and high PD-L1 expression were included in the analysis, of whom 52 received atezolizumab and 73 received pembrolizumab. The mean age of the overall study population was 66.5 years, and it was predominantly male and largely composed of ever-smokers, with the majority of patients presenting with good performance status (ECOG 0–1). Central nervous system metastases were observed in approximately one-fifth of patients (Table 1).

The atezolizumab and pembrolizumab cohorts were generally well balanced with respect to sex, smoking status, age at treatment initiation, PD-L1 tumor proportion score, histological subtype, presence of CNS metastases, and the use of radiotherapy during treatment. A numerically higher proportion of patients treated with pembrolizumab had ECOG performance status 2 compared with those treated with atezolizumab (32.9% vs. 19.2%), although this difference did not reach statistical significance (*p* = 0.106) (Table 2).

In the overall cohort, the objective response rate (ORR) was 25.6%, while the disease control rate (DCR), defined as complete response, partial response, or stable disease, was 69.6%. In the atezolizumab group, the ORR was 19.2% (10/52), with all responses being partial responses. Stable disease was observed in 53.8% of patients, resulting in a DCR of 73.1%, while progressive disease as best response occurred in 26.9% of patients.

In the pembrolizumab group, the ORR was numerically higher at 30.1% (22/73), including complete responses in 4.1% and partial responses in 26.0% of patients; however, this difference in ORR between treatment groups did not reach statistical significance (*p* = 0.214). Stable disease was achieved in 37.0% of patients, yielding a DCR of 67.1%, while progressive disease as best response was documented in 32.9% of patients.

Immune-related adverse events were observed in 40 (32.0%) patients. The most frequent irAEs were thyroid disorders (18.4%), followed by skin toxicity (9.6%), while gastrointestinal, hepatic, pneumonitis, and rheumatologic irAEs were less common (Table 3). Among patients who developed immune-related adverse events, 11 patients (26.2%) experienced more than one irAE, corresponding to 8.8% of the overall study population. Immune-related adverse events occurred in 36.5% of patients in the atezolizumab group and 28.8% of patients in the pembrolizumab group, with no statistically significant difference between treatment arms (*p* = 0.437). Most immune-related adverse events were low grade (CTCAE version 5.0, grade 1–2), while permanent treatment discontinuation due to toxicity was required in two patients both in the pembrolizumab group, including one case of hepatic immune-related adverse event and one case of pneumonitis, both classified as grade 3–4 according to CTCAE version 5.0, with treatment discontinuation performed in accordance with the ESMO Guidelines for the management of toxicities from immunotherapy [13].

**Figure 1:**
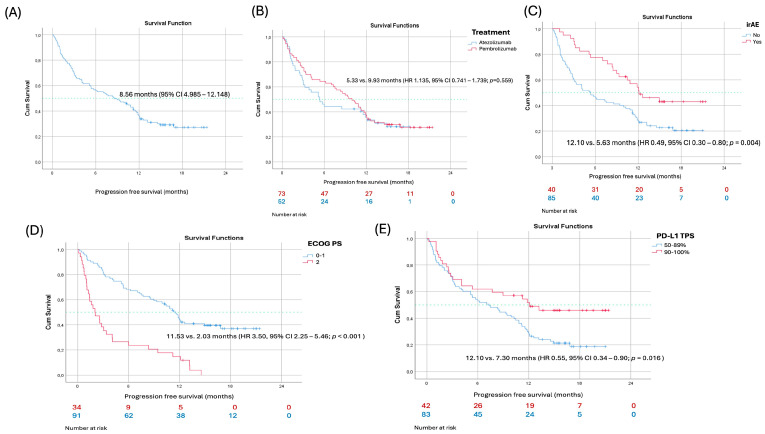
Kaplan–Meier curves for progression-free survival (PFS) in the overall study population (**A**); comparison of PFS between patients treated with atezolizumab and pembrolizumab (**B**); PFS in patients who experienced immune-related adverse events (irAEs) versus those who did not (**C**); PFS according to baseline ECOG performance status (ECOG 0–1 vs. ECOG 2) (**D**); and PFS stratified by PD-L1 tumor proportion score (TPS 50–89% vs. ≥90%) (**E**).

In the overall cohort, at a median follow-up of 15.7 months, the median progression-free survival (PFS) was 8.56 months (95% CI 4.99–12.15). Although the median follow-up was 15.7 months, long-term endpoints such as mature OS remain limited due to the recent reimbursement of atezolizumab for this indication. Patients treated with atezolizumab had a shorter median PFS compared with those treated with pembrolizumab (5.33 vs. 9.93 months); however, this numerical difference did not reach statistical significance (HR 1.14, 95% CI 0.74–1.74; *p* = 0.559) (Figure 1).

In univariable Cox proportional hazards analysis, poor performance status (ECOG PS 2) was strongly associated with inferior progression-free survival (HR 3.50, 95% CI 2.25–5.46; *p* < 0.001). The occurrence of immune-related adverse events was associated with improved PFS (HR 0.49, 95% CI 0.30–0.80; *p* = 0.004), as was high PD-L1 expression (TPS 90–100% vs. 50–89%; HR 0.55, 95% CI 0.34–0.90; *p* = 0.016). Other clinical variables, including sex, age, smoking status, histology, presence of CNS metastases, receipt of radiotherapy, and treatment type (atezolizumab vs. pembrolizumab), were not significantly associated with PFS in univariable analysis.

Variables meeting the predefined significance threshold were entered into the multivariable model. In multivariable analysis, ECOG PS 2 remained independently associated with worse PFS (HR 3.22, 95% CI 2.02–5.12; *p* < 0.001), while high PD-L1 expression retained its favorable prognostic impact (HR 0.57, 95% CI 0.35–0.93; *p* = 0.023). The association between immune-related adverse events and improved PFS was attenuated and did not remain statistically significant after adjustment for other covariates (HR 0.71, 95% CI 0.42–1.18; *p* = 0.180) (Table 4; Figure 1).

## 4. Discussion

In this real-world single-center retrospective analysis of patients with metastatic non-small cell lung cancer and high PD-L1 expression treated with first-line immune checkpoint inhibitor monotherapy, no statistically significant difference in progression-free survival was observed between PD-1 and PD-L1 blockade after adjustment for baseline clinical factors. While a numerical difference in median PFS was noted, treatment type was not independently associated with outcome, suggesting broadly comparable effectiveness of PD-1- and PD-L1-targeting agents in routine clinical practice. These findings are consistent with the current evidence base, as no head-to-head randomized trial has directly compared PD-1 and PD-L1 inhibitors as first-line monotherapy in PD-L1-high metastatic NSCLC. Consequently, available comparative data largely derive from observational cohorts and registry-based studies, which have generally reported similar survival outcomes across PD-1 and PD-L1 inhibitors [14,15,16].

Real-world comparisons of PD-1 and PD-L1 inhibitors in advanced NSCLC, including retrospective analyses, have generally reported no significant differences in progression-free or overall survival between classes of immune checkpoint inhibitors. In a single-center study comparing nivolumab and atezolizumab, PFS and OS outcomes did not differ significantly between treatment groups, suggesting similar effectiveness despite mechanistic distinctions between PD-1 and PD-L1 blockade [15]. In a real-world cohort of elderly patients treated with monotherapy immune checkpoint inhibitors, pembrolizumab and nivolumab exhibited higher response and disease control rates compared with atezolizumab, although overall survival differences were not statistically significant, reflecting the challenges of direct effectiveness comparisons in non-randomized settings [14]. Furthermore, network meta-analyses that estimate relative efficacy across immune checkpoint inhibitors support benefit across PD-1 and PD-L1 agents in patients with high PD-L1 expression, without definitive evidence favoring one over another [16].

Baseline performance status emerged as the most important prognostic factor in this cohort. Patients with ECOG performance status 2 experienced substantially inferior progression-free survival compared with those with ECOG 0–1, and this association remained in multivariable analysis. This finding underscores the central role of functional status in determining outcomes with immune checkpoint inhibitor monotherapy and is consistent with prior real-world observations indicating that patients with impaired performance status derive less durable benefit from immunotherapy [17,18,19,20,21,22,23,24]. Importantly, patients with ECOG PS 2 are frequently underrepresented or excluded from pivotal randomized trials, highlighting the added value of real-world data in characterizing outcomes in this clinically relevant subgroup.

Although patients with impaired performance status represent a substantial proportion of those diagnosed with advanced NSCLC—estimated in some series to account for up to approximately 30%—they remain markedly underrepresented in large first-line immunotherapy trials, emphasizing the value of real-world evidence for assessing treatment outcomes in this clinically important population [24].

The negative prognostic impact of ECOG PS 2 observed in the present study aligns with findings from the phase III IPSOS trial, which specifically evaluated immunotherapy in patients with metastatic NSCLC and poor performance status who were considered ineligible for platinum-based chemotherapy [25]. In IPSOS, outcomes with immune checkpoint inhibition in this vulnerable population were modest, reinforcing the concept that impaired functional status reflects a complex interplay of disease burden, comorbidities, and host factors that may limit the effectiveness of immunotherapy. Taken together, these data suggest that while immune checkpoint inhibitors remain a treatment option for selected patients with ECOG PS 2, expectations regarding treatment benefit should be carefully individualized.

In the present study, very high PD-L1 expression (TPS 90–100%) was independently associated with improved progression-free survival compared with PD-L1 TPS 50–89%, even after adjustment for relevant clinical covariates. This finding is consistent with prior real-world evidence suggesting that increasing levels of PD-L1 expression within the PD-L1-high population may confer incremental benefit from PD-1/PD-L1 axis blockade. In a landmark retrospective analysis by Aguilar et al., patients with metastatic NSCLC and PD-L1 TPS ≥ 90% treated with first-line pembrolizumab demonstrated significantly longer survival outcomes compared with those with PD-L1 expression between 50% and 89% [26]. Similar observations have been reported in other real-world cohorts evaluating first-line immune checkpoint inhibitor monotherapy in PD-L1-high disease, further supporting the reproducibility of this association across clinical settings [18,27,28]. Nevertheless, PD-L1 expression remains an imperfect biomarker. Substantial inter- and intratumoral heterogeneity, variability between primary and metastatic sites, temporal changes in expression, and assay-related differences all limit its precision at the individual patient level [29]. Consequently, while very high PD-L1 expression appears to identify a subgroup with more favorable outcomes, PD-L1 should be regarded as a relative and context-dependent predictor of benefit rather than an absolute determinant of treatment response.

In our cohort, baseline characteristics were numerically less favorable in the pembrolizumab group, which included a higher proportion of patients with ECOG performance status 2 compared with the atezolizumab group (32.9% vs. 19.2%, *p* = 0.106), as well as a lower proportion of patients with very high PD-L1 expression (TPS ≥ 90%) (30.1% vs. 38.5%, *p* = 0.344); both factors associated with poorer outcomes in the overall cohort. Despite these imbalances, patients treated with atezolizumab had a shorter median progression-free survival than those treated with pembrolizumab (5.33 vs. 9.93 months), although this numerical difference did not reach statistical significance (HR 1.14, 95% CI 0.74–1.74; *p* = 0.559). These findings support a cautious interpretation of broadly comparable effectiveness between PD-1 and PD-L1 inhibitor monotherapy in this real-world population. From a practical clinical perspective, these findings support a pragmatic and individualized treatment approach. In patients with PD-L1 TPS ≥ 50% who are candidates for immune checkpoint inhibitor monotherapy, the choice between PD-1 and PD-L1 inhibitors may reasonably be guided by availability, reimbursement policy, toxicity considerations, and clinician experience. Careful assessment of performance status remains essential, particularly in patients with ECOG PS 2, in whom expected benefit may be limited. Furthermore, recognition of very high PD-L1 expression may help refine prognostic expectations and guide patient counseling.

### Limitations

This study has several limitations. First, its retrospective, single-center design is subject to inherent biases, including incomplete data capture and unmeasured confounding. Although data were retrieved from electronic health records, variability in documentation and follow-up cannot be excluded. We acknowledge that immune-related adverse events were analyzed as a time-fixed variable (yes/no), and time-dependent modeling was not feasible due to non-standardized retrospective recording of irAE onset dates; therefore, potential immortal-time bias cannot be fully excluded.

Second, the relatively small sample size limits statistical power, particularly for subgroup analyses, and may have reduced the ability to detect modest differences between atezolizumab and pembrolizumab. (e.g., PD-L1 strata further split by treatment arm)

Third, molecular testing during the study period was limited primarily to EGFR and ALK alterations, as comprehensive genomic profiling was not routinely available, which may have resulted in incomplete exclusion of rare oncogenic drivers. In addition, at the time of analysis, overall survival data were immature due to the relatively short follow-up after the introduction of atezolizumab, and therefore, progression-free survival was used as the primary endpoint. While PFS is a clinically meaningful outcome in this setting, longer follow-up will be required to assess overall survival and confirm the durability of treatment benefit.

Additionally, PD-L1 tumor proportion score and age were analyzed as dichotomized variables despite being continuous by nature, which may have led to loss of information and limited the ability to detect more subtle associations with outcomes. Age was dichotomized using a clinically relevant threshold (≥70 years), consistent with prior real-world immunotherapy studies in elderly NSCLC populations. PD-L1 TPS was categorized as 50–89% versus ≥90% based on previous reports suggesting differential outcomes among patients with very high PD-L1 expression. Moreover, the numerically higher proportion of patients with ECOG PS 2 and the lower proportion of patients with very high PD-L1 expression (TPS ≥ 90%) in the pembrolizumab group may represent a potential source of residual confounding. Although multivariable adjustment was performed, unmeasured or incompletely captured factors may have influenced outcomes. The absence of statistical significance should not be interpreted as proof of equivalence. Given sample size and event numbers, the study may be underpowered to detect modest differences; therefore, findings should be interpreted as suggesting comparable effectiveness within the limits of observational data and available follow-up.

Finally, as a real-world study from a single academic center in a resource-limited setting, treatment patterns, patient characteristics, and access to diagnostics may differ from those in other healthcare systems, potentially limiting the generalizability of the findings.

Despite these limitations, this study reflects routine clinical practice following the introduction of atezolizumab for this indication and provides clinically relevant real-world evidence comparing atezolizumab and pembrolizumab monotherapy in patients with high PD-L1-expressing metastatic NSCLC.

## 5. Conclusions

In this real-world, single-center retrospective study of patients with metastatic non-small cell lung cancer and high PD-L1 expression treated with first-line immune checkpoint inhibitor monotherapy, despite numerical differences, no statistically significant difference in progression-free survival was observed between PD-1 and PD-L1 blockade, with a median progression-free survival of 5.33 months for atezolizumab and 9.93 months for pembrolizumab (HR 1.14, 95% CI 0.74–1.74; *p* = 0.559). Outcomes appeared to be driven primarily by baseline clinical factors.

Poor performance status (ECOG PS 2) was independently associated with inferior progression-free survival, underscoring the critical role of functional status in patient selection for immunotherapy in routine clinical practice. In contrast, very high PD-L1 expression (TPS 90–100%) was associated with improved outcomes, suggesting that gradations within the PD-L1-high population may provide additional prognostic information beyond the conventional ≥50% threshold.

Taken together, these findings support the comparable effectiveness of PD-1 and PD-L1 inhibitor monotherapy in PD-L1-high metastatic NSCLC and highlight the importance of individualized treatment decisions based on patient fitness and tumor biology. Larger, multicenter real-world studies with longer follow-up are warranted to further clarify long-term outcomes and refine prognostic stratification in this setting.

## Figures and Tables

**Table 1:** Baseline characteristics for the whole cohort.

Characteristic	Overall Cohort
Sex	
Male	70 (56.0%)
Female	55 (44.0%)
Smoking status	
Ever smoker	106 (84.8%)
Never smoker	19 (15.2%)
ECOG performance status	
ECOG 0–1	91 (72.8%)
ECOG 2	34 (27.2%)
Age at treatment initiation	
<70 years	75 (60.0%)
≥70 years	50 (40.0%)
Histology	
Non-squamous	95 (76.0%)
Squamous	30 (24.0%)
PD-L1 tumor proportion score	
50–89%	83 (66.4%)
≥90%	42 (33.6%)
Central nervous system metastases at baseline	
Present	24 (19.2%)
Absent	101 (80.8%)
Radiotherapy during immunotherapy	
Yes	34 (27.2%)
No	91 (72.8%)
Any immune-related adverse event	
Yes	40 (32.0%)
No	85 (68.0%)
Treatment group	
Atezolizumab	52 (41.6%)
Pembrolizumab	73 (58.4%)

**Table 2:** Baseline characteristics by treatment group.

Variable	Atezolizumab (*n* = 52)	Pembrolizumab (*n* = 73)	*p* Value
Sex			0.201
Male	33 (63.5%)	37 (50.7%)	
Female	19 (36.5%)	36 (49.3%)	
Smoking status			0.206
Ever smoker	47 (90.4%)	59 (80.8%)	
Never smoker	5 (9.6%)	14 (19.2%)	
ECOG performance status			0.106
ECOG 0–1	42 (80.8%)	49 (67.1%)	
ECOG 2	10 (19.2%)	24 (32.9%)	
CNS metastases			0.071
Present	14 (26.9%)	10 (13.7%)	
Absent	38 (73.1%)	63 (86.3%)	
Age at treatment start			1.000
≥70 years	21 (40.4%)	29 (39.7%)	
<70 years	31 (59.6%)	44 (60.3%)	
PD-L1 TPS			0.344
≥90%	20 (38.5%)	22 (30.1%)	
50–89%	32 (61.5%)	51 (69.9%)	
Any immune-related adverse event			0.437
Yes	19 (36.5%)	21 (28.8%)	
No	33 (63.5%)	52 (71.2%)	
Histology			0.144
Non-squamous	36 (69.2%)	59 (80.8%)	
Squamous	16 (30.8%)	14 (19.2%)	
Radiotherapy during treatment			1.000
Yes	14 (26.9%)	20 (27.4%)	
No	38 (73.1%)	53 (72.6%)	

**Table 3:** Immune-related adverse events in the study population.

irAE Category	n (%)	Atezolizumab	Pembrolizumab
Any irAE	40 (32.0)	19	21
Thyroid irAE	23 (18.4)	14	9
Skin irAE	12 (9.6)	5	7
Gastrointestinal irAE	6 (4.8)	2	4
Hepatic irAE	6 (4.8)	2	4
Pneumonitis	3 (2.4)	1	2
Rheumatologic irAE	3 (2.4)	3	0

**Table 4:** Univariate and multivariate Cox proportional hazards regression analyses for progression-free survival (PFS).

	Univariate Regression Analysis			Multivariate Regression Analysis		
	HR	95% CI	*p*	HR	95% CI	*p*
Sex (male vs. female)	1.294	0.851–1.967	0.227			
Radiotherapy (Yes vs. No)	0.946	0.595–1.504	0.814			
Smoking status (ever vs. never-smoker)	1.112	0.778–1.588	0.560			
ECOG PS (2 vs. 0–1)	3.503	2.248–5.459	<0.001	3.221	2.024–5.124	<0.001
CNS metastases (yes vs. no)	1.510	0.916–2.488	0.106			
irAE (yes vs. no)	0.492	0.303–0.798	0.004	0.705	0.422–1.176	0.180
PD-L1 TPS (90–100% vs. 50–89%)	0.552	0.340–0.895	0.016	0.567	0.348–0.926	0.023
Histology (squamous vs. non-squamous)	1.072	0.656–1.072	0.782			
Age (≥70 years vs. <70 years)	0.842	0.547–1.297	0.436			
Treatment (atezolizumab vs. pembrolizumab)	1.135	0.741–1.739	0.559			

## Data Availability

The dataset generated and analyzed during the current study is not publicly available due to privacy regulations and consent restrictions but is available from the corresponding author upon reasonable request. All data shared will be de-identified in accordance with ethical guidelines.
